# From the Problem of Corrosion to Green Solutions: The Role of Biosurfactants as Anti-Corrosion Agents

**DOI:** 10.3390/ma19040743

**Published:** 2026-02-14

**Authors:** Kaio Wêdann de Oliveira, Yslla Emanuelly da Silva Faccioli, Gleice Paula de Araújo, Attilio Converti, Rita de Cássia Freire Soares da Silva, Leonie Asfora Sarubbo

**Affiliations:** 1 Instituto Avançado de Tecnologia e Inovação (IATI), Rua Potyra, n. 31, Prado, Recife 50751-310, Pernambuco, Brazil; kaio.olliver@hotmail.com (K.W.d.O.); ysfaccioli@hotmail.com (Y.E.d.S.F.); gleice.araujo@iati.org.br (G.P.d.A.); rita.freire@iati.org.br (R.d.C.F.S.d.S.); 2 Rede Nordeste de Biotecnologia (RENORBIO), Universidade Federal Rural Pernambuco (UFRPE), Rua Dom Manuel de Medeiros, s/n, Dois Irmãos, Recife 52171-900, Pernambuco, Brazil; 3 Department of Civil, Chemical and Environmental Engineering, Pole of Chemical Engineering, Via Opera Pia 15, 16145 Genoa, Italy; 4 Escola de Tecnologia e Comunicação, Universidade Católica de Pernambuco (UNICAP), Rua do Príncipe, n. 526, Boa Vista, Recife 50050-900, Pernambuco, Brazil

**Keywords:** biosurfactants, corrosion, green inhibitors, sustainability, green chemistry

## Abstract

Corrosion remains one of the major contemporary technological challenges, causing significant economic, environmental, and operational impacts on industrial systems. Although it is a spontaneous process inherent to metals and their alloys, its progression can be significantly mitigated by appropriate protection strategies. Traditionally, synthetic inhibitors have been widely used; however, their toxicity, environmental persistence, and increasing regulatory restrictions have prompted a search for greener alternatives. Biosurfactants stand out as promising green anticorrosive agents, acting through the formation of adsorbed films, reduction in wettability, modification of the metal–medium interface, and, in some cases, antimicrobial effects that inhibit the formation of corrosive biofilms. This review presents an integrated analysis of the main corrosion mechanisms, including uniform, localized, galvanic, and microbiologically influenced corrosion, with an emphasis on critical industrial environments such as the maritime, petrochemical, energy, and infrastructure sectors. Additionally, the main classes of biosurfactants are discussed, along with their key physical and chemical characteristics, including critical micelle concentration, thermal and saline stability, adsorption capacity, and their mechanisms of action in mitigating corrosion. Finally, the article summarizes the advances of the last decade, highlighting experimental studies, emerging applications, and technological trends that consolidate biosurfactants as viable, efficient, and environmentally safe alternatives for industrial corrosion protection.

## 1. Introduction

Corrosion is a serious issue, impacting several industrial sectors globally and leading to significant economic damage. It consists of the deterioration of a material due to its interaction with the environment, which can happen at any time [1,2]. Although this definition applies to all materials, it is most used for metals and metal alloys. Additionally, corrosion not only alters the chemical characteristics of the material but also changes its physical and mechanical characteristics, directly threatening the structural integrity of equipment and infrastructure [3,4].

Corrosion of metals, especially iron and steel, causes significant economic and operational losses and affects several other metals [5,6]. Its impacts include fluid or gas leaks, decreased mechanical strength, and, in severe cases, structural failure, which compromise operational safety and the environment. This phenomenon damages industries such as civil construction, petrochemical refining, mining, fertilizer production, and energy generation, leading to equipment failures, shutdowns, toxic leaks, and high maintenance and replacement costs [7].

According to 2016 data from NACE (National Association of Corrosion Engineers) International, in 2013, the estimated global cost of corrosion was US$2.5 trillion annually, equivalent to 3.8% of global GDP. In Europe, the overall corrosion costs for 2013 were estimated at US$701.5 billion. Distribution of corrosion-related costs in the USA is provided. Many countries expect that corrosion costs will vary from 1% to 5% of GDP each year [8,9]. Implementing already-available effective corrosion control techniques could reduce costs by 15 to 35%, leading to global yearly savings of US$375–875 billion [10].

Among various corrosion phenomena, Microbiologically Influenced Corrosion (MIC) has gained increasing attention due to its significant economic and operational impacts. MIC is the deterioration of metals and materials caused or sped up by the activity of microbial cells that attach to metallic surfaces. This phenomenon accounts for hundreds of millions of dollars in losses each year worldwide, especially in sectors of oil and gas, marine infrastructure, water distribution, and wastewater treatment [11,12].

Although corrosion is highly common and occurs in many forms, making its complete elimination and the related costs impossible, several studies show that 25 to 30% of the global annual costs linked to corrosion could be avoided by adopting optimized corrosion management practices [13,14]. In this way, proper protection, monitoring, and control methods could significantly reduce the damaging effects, prevent premature failures, and lower environmental risks, as well as the costs of maintenance, repair, and replacement of metallic materials.

Historically, corrosion mitigation has depended heavily on the widespread use of synthetic inhibitors, mostly made from organic and inorganic compounds that contain amines, phosphates, chromates, and nitrites. They are frequently applied in sectors such as machinery, refining, petroleum, civil construction, chemicals, and energy because they are effective, simple, and inexpensive. Although they work well, many of these synthetic inhibitors pose environmental and health risks, and stricter regulations are increasing, prompting greater interest in sustainable and environmentally friendly alternatives [15,16].

In this context, biosurfactants are important for preventing metal corrosion due to their unique properties and sustainability. Also called bio-based or green surfactants, they are produced by yeasts, fungi, and some bacterial species [17,18,19]. They have a wide range of chemical structures, including lipopeptides, glycolipids, phospholipids, and proteins, making them suitable for several industrial uses due to their structural diversity and functional properties. Biosurfactants are utilized in areas like food, agriculture, petroleum, and cosmetics because they lower surface and internal tension and have emulsifying, stabilizing, wetting, spreading, foaming, and cleaning abilities similar to those of chemical surfactants [20,21,22].

In aqueous corrosive environments, metal dissolution leads to the formation of metal cations (e.g., Fe^2+^ and Fe^3+^) at metal/solution interface. Biosurfactants with functional groups such as C=O, −OH, −NH, −COOH, and −PO_4_^3−^ possess heteroatoms capable of interacting with these cations through electrostatic interactions and coordination bonding. This interaction promotes biosurfactant adsorption on metal surface, leading to formation of a protective film capable of hindering anode and cathode reactions. In systems involving microbiologically influenced corrosion, such groups can also interact with biofilm components, reducing biofilm adhesion and altering surface wettability, thereby helping to decrease corrosion [20].

Given the significant economic, structural, and environmental impacts associated with corrosion, as well as the limitations and disadvantages of synthetic inhibitors—including their potential toxicity, low biodegradability, increasing regulatory restrictions, and decreased performance under harsh environmental conditions like high salinity, high temperature, and microbiologically influenced corrosion—biosurfactants stood out as emerging anti-corrosion compounds. They are valued for their proven inhibitory effectiveness and, often, their lower environmental impact compared to traditional synthetic inhibitors [15]. This review critically analyzes corrosion across various industrial contexts and provides an updated overview of the utilization of biosurfactants as sustainable anticorrosive agents, discussing their main classes, physicochemical properties, and mechanisms of action. Finally, challenges, trends, and future perspectives regarding the adoption of these biocompounds as sustainable alternatives in industrial corrosion protection are explored.

## 2. Corrosion

Corrosion processes are mainly electrochemical, involving coupled anode and cathode reactions at metal/environment interfaces. In some environments, corrosion has traditionally been described as chemical; however, electrochemical principles control most corrosion mechanisms [23]. Corrosion begins through various processes that cause changes in the metal surface and local environment, such as oxide formation, pH shifts, redox potential changes, and transfer of metallic ions into covering matrices [24].

**Figure 1:**
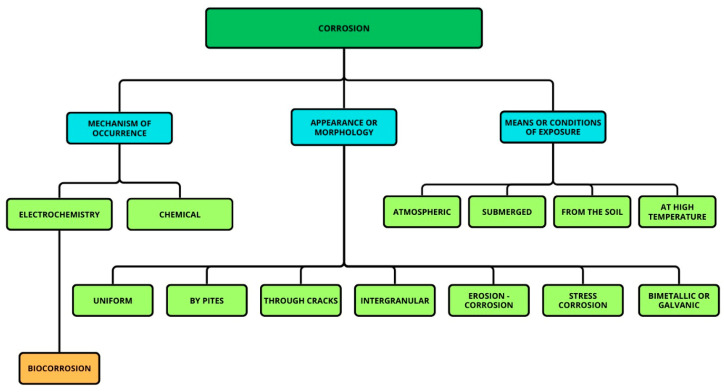
Flowchart depicting the mechanisms of corrosion occurrence, their morphologies, and their exposure conditions or environments.

Corrosion processes are classified as chemical or electrochemical based on the environment’s characteristics. They are also categorized by the surface morphology of the corroded materials or the causes that promote corrosion [25]. In the flowchart shown in Figure 1, we can observe the mechanisms of occurrence, the types of morphology, and the conditions that lead to corrosion.

### 2.1. Mechanisms of Occurrence

#### 2.1.1. Electrochemical Corrosion

Electrochemistry is the science that studies chemical reactions involving the transfer of electrons at the interface between two conductors, one electrical (electrode) and one ionic (electrolyte), which is a solution that conducts electricity due to the movement of ions [26]. When electrochemical reactions occur on a metallic surface, metal deterioration results from anodic dissolution processes that are necessarily coupled with simultaneous cathode reductions, including oxygen reduction or hydrogen formation, in order to maintain electrochemical equilibrium. This means that a metal (M) becomes a metallic ion with a positive electrical charge (M^+^) [27].

**Figure 2:**
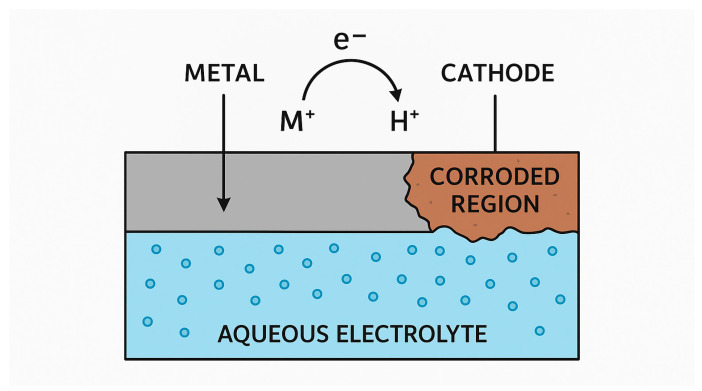
Diagram of electron transfer from the metal to the corrosion region.

Electrochemical corrosion of metals occurs in an electrolytic environment, driven by the metal’s electrochemical properties (standard electrode potential, E^0^) and the electrolyte composition. The environment typically directly influences the corrosion behavior of metals. Within the electrolyte, differences in structure and composition among different parts of the metallic materials can create potential differences and initiate corrosion. Acidic and oxygen-rich electrolytes are more prone to cause electrochemical metal corrosion [24]. Figure 2 shows the electron exchange that occurs during corrosion.

Corrosion involves anode-to-cathode electron transfer in an electroconductive medium. The metal at the anode releases free electrons (oxidation), while the cathodic metal accepts electrons (reduction). The metal with the lower (more negative) reduction potential functions as the anode and undergoes corrosion, while that with the higher reduction potential acts as the cathode. To allow corrosion, the following conditions are needed: 1. Separate cathode and anode regions are required to allow current flowing through the conductive solution. 2. Oxidation occurs at the anode, where the metal surface is depleted as it reacts with oxygen, leading to corrosion. 3. Reduction takes place at the cathode, where dissolved oxygen serves as an electron acceptor, being reduced to hydroxyl ions (OH^−^) in neutral or alkaline media, or to water in acidic environments [28].

Indeed, metal corrosion consists of a redox reaction where oxidation and reduction semi-reactions take place at the anode and cathode sites on metal surface. Electrons resulting from metal or low-valence metal ion anode oxidation reach the cathode via conductive metal substrates, which react with protons, oxygen gas, or other reagents to end the reaction. The surrounding environment, especially the pH [29], greatly influences metal corrosion.

From an electrochemical perspective, MIC does not constitute a separate corrosion mechanism but rather an alteration of traditional electrochemical corrosion processes, where microbial activity changes interfacial reactions, mass transport, and local redox conditions at the metal/electrolyte interface [11].

**Figure 3:**
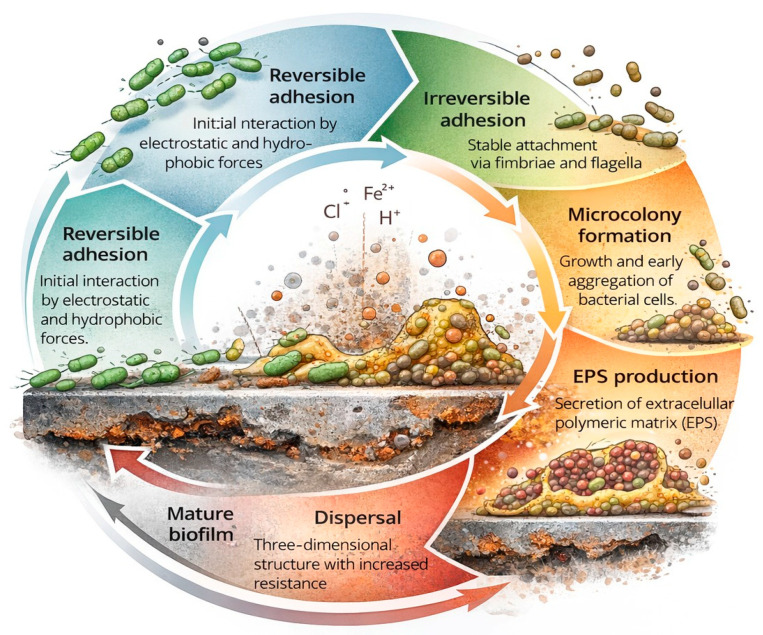
Biofilm formation process.

In MIC processes, microorganisms secrete extracellular polymeric substances (EPS), which promote adhesion to metal surfaces and help form biofilms (Figure 3). The interaction between biofilms, EPS, and metal surfaces can change local electrochemical conditions, increase metal dissolution, and weaken protective passive layers. Several groups of microorganisms have been linked to MIC, including sulfate-reducing prokaryotes, iron- and manganese-oxidizing bacteria, iron- and nitrate-reducing bacteria, methanogenic archaea, and halophilic microorganisms [12,30].

Biofilms are often responsible for MIC, an electrochemical process. The primary form of corrosion in steel is pitting corrosion because some MIC products, like iron sulfide, are partially passivating and semiconducting, which can result in a large cathode and small anode setup [31,32].

#### 2.1.2. Chemical Corrosion

Chemical corrosion occurs through direct reactions between metallic structures and corrosive gases in the absence of moisture. It is driven by thermodynamic forces and oxidation kinetics, as it does not involve the formation of electrochemical cells, unlike wet corrosion [33]. The protection capability of the corrosion product layer largely depends on its physical and mechanical properties, which can be assessed using the Pilling-Bedworth ratio—which is widely used to evaluate the compactness of an oxide film on an alloy surface, since it considers the volume change associated with forming a passive film on a metal [34,35]. For instance, aluminum readily reacts with O_2_ to create a compact Al_2_O_3_ layer, leading to passivation rather than harmful corrosion. Therefore, understanding chemical corrosion requires evaluating both the thermodynamic stability and the characteristics of the formed oxide layer [36].

### 2.2. Corrosion Mechanisms

Mechanically assisted corrosion involves degradation processes driven by the additive effects of physical forces and electrochemical reactions. Unlike corrosion that is judged solely by surface appearance, these processes are influenced by stress, flow conditions, and material deformation, which greatly speed up electrochemical dissolution and material failure [37].

#### 2.2.1. Erosion Corrosion

Erosion corrosion (E-C) is a degradation process involving both electrochemical corrosion and wear mechanisms, most common in industrial equipment and components that handle liquid flows [38,39]. During E-C, the mass loss observed exceeds the combined losses from plain erosion and corrosion occurring independently. Interplay between these two processes has been described by various researchers as a ‘synergistic’ effect [40].

E-C significantly influences various production sectors, such as the maritime, petroleum and gas, atomic, high-temperature, electricity production, extractive, and processing ones. Additionally, the impact of E-C on the dental, food, and aeronautical sectors cannot be overlooked [41,42].

#### 2.2.2. Stress Corrosion Cracking (SSC)

SSC is a degradation process driven by the joined effect of mechanical stress and electrochemical corrosion, which appears as crack initiation and growth. Diverse kinds of mechanical stress in corroding environments, including vibrations, tensile stress, and arc stress, lead to faster crack development in a vulnerable and likely failing material [43,44].

It is a localized failure, which is more severe under the synergistic effects of stress and corrosion than would be expected if the two effects were simply added together. Many variables influence the initiation of SCC, including alloy composition, corrosive environment, temperature, and time. There are methods to relieve internal stress that can reduce the susceptibility of materials to SCC using fracture mechanics [45].

### 2.3. Morphology or Appearance of Corrosion Type

Although corrosion types are often classified by their surface morphology or appearance, many of these phenomena are driven by different electrochemical or physicochemical mechanisms [46]. In this section, corrosion is described based on its characteristic morphological features, clearly connecting each type to its underlying theoretical mechanism. This distinction is crucial to prevent conceptual confusion between corrosion mechanisms and their observable effects.

#### 2.3.1. Uniform Corrosion

Uniform corrosion (UC) causes the regular loss of metal from a corrosive surface, leading to a general reduction in wall thickness [47]. Considered as the prevalent and plainest type of metallic corrosion, it results from a uniform attack on the outer layer caused by corroding environments. Metal solubilization is caused by chemical attacks or metal conversion into metal ions, involving oxidation. At high temperature, UC happens when the metal reacts with other corroding compounds, including oxygen, to form oxides. Zinc solubilization in HCl provides an example of such a corrosion kind [48].

#### 2.3.2. Bimetallic or Galvanic Corrosion

Galvanic corrosion is an electrochemical reaction involving a galvanic couple and an electrolyte, which results in accelerated oxidation of the less noble metal [49]. It occurs when two different metals, placed in contact with each other, are submerged in corroding media, such as a corrosive liquid. A current flows from the metal with the lower standard electrode potential (anode) to the one with the higher potential (cathode), causing increased corrosion at the anode while preventing oxidation at the cathode [50,51,52].

#### 2.3.3. Pitting Corrosion (PC)

PC is a type of localized corrosion caused by breakdown of passive films and highly localized electrochemical reactions, appearing as small cavities or pits on metal surface [53,54].

Pitting formation is complex and poorly understood, but it is believed to involve the disruption of the passivation layer. Copper is a passivating metal that, when oxidants are present, forms an incrustation layer on its surface, thereby limiting corrosion and protecting the metal from further chemical attack. It is believed that pitting in many metals begins with a defect in the passivation layer, leading to localized corrosion [55].

Some corrosion pits, formed by the breakdown of a protective passive film, can exhibit intricate shapes, which reveal valuable information about the pit itself. They exhibit a wide variety of shapes, including shallow, deep, elliptical, dish-shaped, etched, recessed, narrow, subsurface, polished, wide, occluded, hemispherical, and crystallographic. The wide variety of pit shapes observed is therefore a consequence of the localized electrochemical conditions established during pit initiation and propagation [56].

#### 2.3.4. Crevice Corrosion

Crevice corrosion (CC) is driven by electrochemical potential differences caused by differential aeration, resulting in localized corrosion within occluded regions. It happens in areas of materials in contact with stagnant corroding fluids. The reaction is triggered by corroding substances present on metal surface [57]. The obstructed area develops highly destructive chemistry, creating an environment that favors metal solubilization within the crack. Redox potential difference causes a selective leaching by CC or PC. Cracks can form between two metals or between one metal and one nonmetal surface [58,59].

This type of corrosion features a geometric setup where the cathodic reagent (such as oxygen) can easily reach metal surface outside the crack through convection and diffusion, while access to the stagnant solution inside the crevice is much more difficult and can only occur through diffusion via the narrow opening. Therefore, potential differences between the exposed metal surface and those within the crevices trigger the operation of corrosion cells, known as “oxygen concentration cell action” [60,61].

An electrochemical reaction in CC involves metal (M) oxidation and reduction of an oxidant present in aqueous solution, such as oxygen, to hydroxide ion:

(1) Oxidation: M → M*^n^*^+^ + *n* e^−^
(2) Reduction: O_2_ + 2 H_2_O + 4 e^−^ → 4 OH^−^

The anodic reaction produces an abundance of metallic cations that can promote the hydrolysis of the metallic cation:

(3) *M^n^*^+^ + H_2_O → *M*OH^(*n*−1)+^ + H^+^

This overall reaction produces protons, which cause a decrease in pH in the crevice and forms the basis of the acidification mechanism of crevice corrosion. Initially, these two reactions occur simultaneously across the entire surface, including the crevice, in the same manner as general corrosion [61].

#### 2.3.5. Intergranular Corrosion (IGC)

IGC is a phenomenon whose precise mechanisms have been debated for almost half a century. It is a special form of corrosion influenced by microstructure, in which the grain boundary “region” of the alloy is electrochemically different from the microstructure of the adjacent alloy or in bulk [62,63,64].

**Figure 4:**
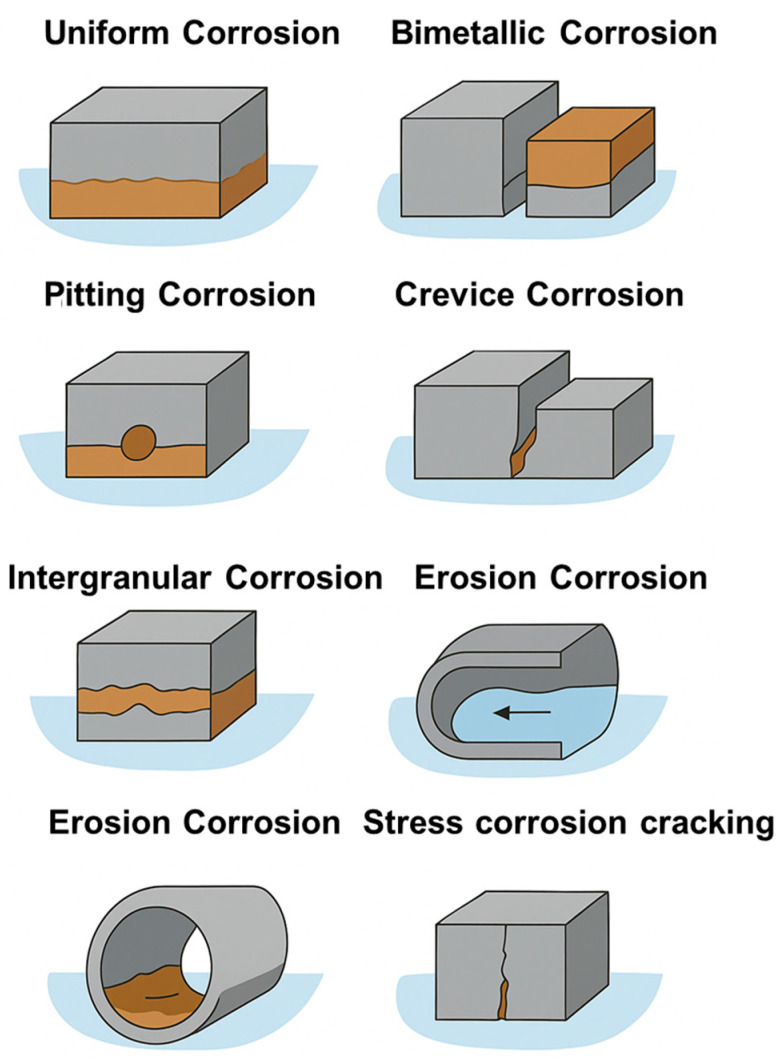
Schematic illustration of the main kinds of corrosion in metals.

One of the primary causes of intergranular corrosion is sensitization, which mainly occurs in 18Cr-8Ni (austenitic) stainless steels due to improper heat treatment. This heat treatment decreases chromium levels near grain boundaries, making the alloy close in composition to common nickel-containing steel in those regions. As a result, sensitization at the grain boundaries creates a less corrosion-resistant and more anodic alloy, leading to intergranular corrosive attack. This kind of corrosion is driven by the potential difference between cathodic grains and anodic regions of grain boundaries [65]. Figure 4 schematically illustrates the different kinds of corrosion and their specific characteristics.

Several studies have aimed to understand these phenomena, emphasizing details about the type of corrosion, experimental conditions, and investigated objectives. Table 1 provides a summary of examples from the literature, related to the main types of corrosion, the material or environment studied, and the goals set by the authors.

The above synthesis highlights the diversity of approaches adopted for the study of corrosion, emphasizing that the choice of material and environment directly influences the observed mechanisms. This variety of scenarios is presented in the following section, as well as the justification and the search for more efficient and sustainable mitigation measures, including the utilization of inhibitors and biosurfactants.

### 2.4. Industrial Segments and Corrosion Process

Like other natural degradation processes, such as earthquakes or severe weather events, corrosion can cause dangerous and costly damage to a wide range of critical infrastructures, including oil pipelines, bridges, public buildings, vehicles, and water and sewage systems. These sectors are some of the most affected ones, where corrosion directly compromises structural integrity, operational safety, and economic performance [13]. Below, the main industries covered in this review and their exposure to corrosion-related degradation are discussed.

#### 2.4.1. Petroleum, Gas and Petrochemical Sectors

In the petroleum and gas sector, corrosion-related failures account for over 25% of all safety incidents. Corrosion in oilfield environments mainly results from acid gases dissolved in oilfield brine, including CO_2_ (leading to sweet corrosion) and H_2_S (causing acid corrosion); O_2_ corrosion occurs in waterflooding oil tanks, other tank components, and metal parts with some manufacturing flaw [80,81].

Given the unique conditions of oil refinery operations, some types of corrosion are specific to this equipment, including polythionic acid, sulfide, H_2_, acid water, CO_2_/petroleum/fuel, and amine solution corrosions [82].

In oil and chemical processing plants, corrosion under insulation (CUI) poses a major challenge for material integrity; it also results in increased costs for equipment maintenance and replacement. Its main cause is water or condensed humidity penetrating the insulating material, enabling moisture to diffuse through it. This moisture then makes direct contact with the metal beneath, leading to CUI [83,84].

Statistics from ExxonMobil show that CUI can account for 40 to 60% of pipeline operating costs. For carbon steel, its rate can be as much as 20 times greater than that of corrosion in naturally ventilated environments. If left unaddressed, CUI can have serious consequences [84].

Sanni et al. [85], who examined the corrosion effect in oil wells, noted that carbon steel is subject to corrosion, especially in acidic environment. Various acids, including acetic acid, HCl, and HF, are commonly used for scraping, pickling, acidic cleaning, and acidification in petroleum sector. Jin et al. [86] observed that, among the many failure situations, most heat exchanger failures are caused by corrosion. Due to the high-speed flow of corrosive components through the heat exchanger bundle, continuous erosion has led to a multidimensional, multifaceted corrosion mechanism. Although refining companies have invested significantly in corrosion control and process protection, losses associated with flow-related corrosion mechanisms, such as erosion-corrosion and flow-accelerated corrosion (FAC), have continued to rise. This trend is linked to higher flow velocities, more severe operating conditions, and aging industrial infrastructure, all of which accelerate metal deterioration despite preventive measures [87]. Therefore, effectively solving the problem of heat exchanger flow corrosion is an important issue for the development of the petrochemical industry and the safe operation of process equipment.

Corrosion is the leading factor responsible for 46.6 and 70.7% breakdowns in natural gas and crude petroleum pipelines, respectively. An estimate of corrosion costs made by a well-known petroleum and gas company showed that they reached about US$900 million in 2003. Global spending on corrosion in petroleum and gas sector is roughly US$60 billion, of which US$1.372 billion in USA. Additionally, with growing energy demand from petroleum and gas and related worry, industrial corrosion costs are expected to keep rising globally [88].

Jasim et al. [89] emphasized that corrosion issues in the petroleum sector should be addressed by taking into consideration several items. The acids employed in stimulation, the origin and kind of tank materials, and the petroleum well facilities, with operating conditions depending on coatings and pipes, are among the factors that affect corrosion. Therefore, it is necessary to examine each case individually to reach a definitive decision on innovative materials.

#### 2.4.2. Shipbuilding and Maritime Industry

Maritime transport is a crucial driving force of the globalized economy today, and its role greatly influences consumer prices, global warming, and marine pollution. So, it is essential to tackle the issues that commonly threaten this sector, including corrosion [90]. In shipbuilding, maritime structures are heavily harmed by corrosion, compromising mechanical features and various structural components, like hull structural failure. Statistical analyses indicate that corrosion accounts for about 90% of the costs related to ship structural defeats [91].

Ships are especially vulnerable to corrosion because of contact with seawater, atmosphere, and corrosive materials. Metal corrosion in seawater happens faster than in freshwater due to the high electrical conductivity and salts present in seawater. Currently, measuring the reduction in tin thickness on ship hulls is performed using ultrasonic devices and electronic data processing, even through paint layers. Additionally, modern corrosion detection methods that depend on sensors are commonly used [92].

Focusing on the types of corrosion on ships, general corrosion commonly affects larger areas, such as the hull, while localized corrosion, such as pitting and groove corrosion, tends to occur in small components, including pipes, filters, and valves. Stowage tanks are essential to proper handling of the ship as they provide ballast for ship stabilization and water for inner seawater facilities, including fire fighting, engine heat management, climatization, and freshwater production [93].

Vourna et al. [94], evaluating corrosion in marine steel immersed in saltwater with a nondestructive magnetic sensor, demonstrated that DH36 marine steel corrosion proceeds through different morphological and electrochemical stages. Starting exposure caused quick metal solubilization and formation of weekly adhered lepidocrocite and goethite, offering poor barrier protection. Extended submersion resulted in the formation of dense and a layered corrosion morphology, along with microcracks and increased porosity.

In their research, Buruiana et al. [95], studying the corrosive behavior of two types of naval steel using electrochemical and gravimetric methods, namely grade A steels, which are moderate-strength structural carbon steels, and E36 steel, a higher-mechanical-strength structural steel, immersed in a solution of 0.4% ammonium sulfate and 0.05% sodium chloride, concluded that the dissolution process begins with localized pitting corrosion and that the rate of E36 steel corrosion is significantly lower than the one of grade A steel. The harsh reality of the marine environment, including cyclic mechanical stresses, saline conditions, fluctuating seawater pH levels, and marine biofouling, highlights the need to develop robust and durable materials to meet the challenges posed by ship corrosion, from surface corrosion to microbial degradation [96].

#### 2.4.3. Power Generation Industry (Solar, Nuclear and Wind)

Solar energy is a widely available, sustainable, and renewable energy source. As a renewable resource, solar energy has the potential to soon replace common fossil fuels [97,98]. Solar panels (photovoltaic modules) are vital for converting sunlight into electricity, but their long-term efficiency can be compromised by corrosion, which results from various degradation mechanisms. This process reduces light absorption by the cells and, consequently, the efficiency of energy conversion, making the study of corrosion fundamental to assure the durability and performance of solar panels [99,100,101,102]. Electrochemical corrosion, the most frequent and subtle degradation process impacting on photovoltaic modules, consists of redox reactions between the metallic contacts of solar cells and surroundings. Moisture and temperature variations help form electrochemical cells on the surfaces of solar cells, leading, over time, to pitting corrosion or general deterioration of the material [103]. Assuming rises in oil and natural gas prices, along with significant environmental pressures from coal use, nuclear power becomes an inevitable strategic alternative for more sustainable development. Atomic power stations, which allow producing large amounts of electricity using only a few nuclear fuels [104,105], utilize valves as key devices that, however, undergo considerable deterioration. 304 Stainless steel is commonly employed as a seal face material for these valves because of its special qualities, such as outstanding elasticity, strength, and weldability. Wear and corrosion can damage their sealing surfaces during extended service [106,107].

FAC in atomic power station pipelines is a major cause of accidents, fatalities, damage, and disruptions. Pipe ruptures caused by wall thinning from corrosion are the leading cause of accidents in nuclear power plants. One of the most vulnerable locations to wall thinning from FAC is at (or just downstream of) pipeline elbows. This is because the change in flow direction causes oxide layers to accumulate on the inner surfaces of the pipes, leading to corrosion [108].

The use of offshore wind energy as a new energy source can lower greenhouse gas emissions and air pollution to combat climate change, thus promoting a sustainable energy system and enhancing global energy security [109,110,111]. However, the marine environment is complex, with variable seafloor movement and environmental loads such as waves, currents, and winds that significantly impact marine engineering structures. Because of the marine environment, steel pipe pile foundations are in direct contact with seawater, facing serious corrosion issues [112].

Corrosion on the surface of steel pipe piles changes their surface structure, reduces the thickness of components, and can alter shear stress at the pile–soil interface, potentially weakening the bearing capacity of pile foundations and jeopardizing structural safety. Pitting corrosion causes especially severe damage, creating local stress concentrations in the steel pipe pile, which affects the pile’s service life and accelerates the loss of its bearing capacity. The extent of corrosion depends on environmental factors, including salt content, pH, temperature, wave activity, among others [113].

#### 2.4.4. Construction Industry

Reinforced concrete (RC) serves as the basis of contemporary infrastructure, being widely used in buildings, bridges, and maritime constructions because of its extraordinary mechanical features and economic efficiency. In the last two decades, many RC structures, such as road/rail bridges, skyscrapers, and power plants, have been built with a target service life of over 100 years. Many of these RC structures are exposed to highly aggressive environments; therefore, to achieve this goal, the steel–cement systems in these structures must have good corrosion resistance [114,115]

Corrosion of concrete reinforcement is one of the most common deterioration mechanisms found in RC structures, affecting both their durability and structural capacity. The corrosive process weakens reinforcement strength and ductility, in addition to worsening fatigue resistance and bond strength. Consequently, structure mechanical properties and operability are impaired, with signs typically including severe cracking, large deflections, and even brittle failure [116]. The steel corrosion process within concrete is an electrochemical reaction that occurs when O_2_ and humidity are present. The primary mechanisms include chloride-induced corrosion, sulfate attack, and carbonation-induced corrosion [117]. When oxygen and moisture infiltrate the surrounding concrete, the steel reinforcement corrodes. The corrosion products occupy a larger volume than the original steel, exerting internal pressure on the reinforcement, thereby inducing tensile stresses in concrete and impacting structural performance [118,119].

Concrete corrosion is mainly due to chloride ions penetrating concrete, especially in a corrosive environment. After these ions breach concrete layer and achieve steel reinforcement, rust forms. This causes the steel to expand, leading to further concrete cracking and spalling and contracting reinforcement effective cross-sectional area [120].

Qian et al. [121], when evaluating atmospheric chloride-induced corrosion in a reinforced concrete beam in contact with a real marine environment for 7 years, observed that the first peak of chloride concentration appeared at an internal migration depth of 3 mm to 5 mm, influenced by environmental changes affecting the concrete surface. They also noted that chloride corrosion in the concrete beam was impacted by carbonation.

In their review of the role of cracks in chloride-induced corrosion of carbon steel in concrete, Poursaee and Ross [122] concluded that cracks have a negative impact on concrete durability and influence chloride penetration and chloride-induced corrosion in the reinforcement in terms of initiation and propagation phases. Therefore, when cracks are narrow, corrosion is delayed, likely because of self-repair and corrosion products that fill the cracks, thereby hindering access to O_2_ and chloride ions.

According to Klein et al. [123], who evaluated the influence of sulfate on corrosion of concrete-reinforcing steel, choosing the right construction site for the low-level radioactive waste repository is essential to meet the facility’s durability requirements. Among many factors, the structure’s durability depends on the presence of sulfate in the soil and/or groundwater at the site. Specifically, the presence of sulfate alone increases the corrosion rate of reinforcing steel bars, and the combined use of sulfate and chloride further amplifies the susceptibility to corrosive attack compared to chloride alone. Both ions act independently, and the corrosion rate is equal to that of the two effects added together.

Kanagaraj et al. [124] observed a deterioration in the functional capacity of geopolymer concrete beams when they were exposed to high temperature and corrosion. In particular, the joined effects of corrosion and fire had a negative impact on final load-bearing capacity, plasticity, rigidity, bending toughness, ductility, and energy absorbing capacity.

When reinforcements corrode, rust formation causes a loss of bonding between steel and concrete, leading to delamination and spalling, which can ultimately result in structural failure [125]. In United States, the direct yearly cost of highway bridge corrosion was approximately US$8.3 billion in 2014. The American Society of Civil Engineers projected that infrastructure repairs would cost around US$2 trillion by 2025. In China, the cost of reinforcement corrosion was approximately 1.2% of GDP, exceeding US$2 billion in 2021 [126].

### 2.5. Corrosion Inhibitors

Corrosion inhibitors are chemicals added in relatively low or moderate concentrations, typically ranging from a few tens to several thousand ppm, depending on the inhibitor formulation, the corrosive environment, and the operating conditions, to prevent or slow down corrosion without significant reactions with environmental components. Corrosion inhibitors are very important in sectors including oil extraction, processing, and chemical industries. The addition of corrosion inhibitors to a structure retards corrosion or reduces metal oxidation rate. Inhibition is the process that hinders the attack of corroding agents by adsorption of inhibitors throughout the metallic substrate [127,128,129].

Corrosion inhibitors are substances specifically added to environments or materials to decelerate corrosion. They act by disrupting corrosion through different mechanisms, namely, creating a protective surface layer, changing electrochemical reactions, or removing corrosive species [130]. Corrosion inhibitors are very important practically because they are widely used to reduce metallic waste during production and to lower the risk of material failure, which can cause sudden shutdowns of industrial processes and lead to additional expenses. It is also crucial to utilize corrosion inhibitors to prevent mineral dissolution and decrease consumption of acids [49].

There are two kinds of corrosion inhibitors based on their origin: natural and synthetic inhibitors. Synthetic inhibitors, including amine salts, phosphates, and nitrites, are produced to offer high effectiveness in various corrosive environments. Although these substances are more efficient, have a broader applicability, and last longer than natural counterparts, they are more costly and need careful selection and dosing to prevent environmental damage and interference with other chemical processes in the system [131,132].

On the other hand, natural inhibitors, derived from plant or mineral sources, stand out for being biodegradable, green, and having low toxicity. Common examples are vegetable oils, tannins, and salicylic acid. Despite these benefits, their effectiveness can be limited in highly corrosive environments, and in some cases, they are more expensive than synthetic inhibitors. Nevertheless, these compounds are becoming more relevant as sustainable alternatives, utilized in the petroleum and gas sector, in the preservation of cultural artifacts, and, because of their safety and renewable nature, in sensitive sectors like food and pharmaceuticals [133,134].

Corrosion inhibitors are chemicals capable of interacting with the surface of materials or modifying environs to significantly improve their resistance to corrosion in a specific setting. They can be categorized by their chemical makeup as organic, inorganic, or green/biological, or based on their inhibition mechanism, either those that disrupt or slow down anode oxidation and/or cathode reduction, or those that form a protecting layer or film on outer metal surface. Therefore, inhibitors can be grouped as anodic, cathodic, or mixed inhibitors. Nonetheless, the most common classification system is based on chemical composition [135].

#### 2.5.1. Organic Corrosion Inhibitors

Organic corrosion inhibitors are often utilized in industrial applications as they are effective over a broad temperature range, compatible with protected materials, moderate in toxicity, and highly soluble. Effective organic corrosion inhibitors require heteroatoms with lone pairs of electrons, such as the ones found in N, O, S, and P, along with electron-rich groups like benzene rings and multiple bonds capable of interacting with a metal’s unoccupied *d* orbital and promoting adsorption [136].

The effectiveness of an organic inhibitor depends on factors like the size, aromaticity, kind and number of atoms or bonds (π or σ), surface charge, molecule charge density, and nature of corrosive environment. Molecule adsorption on metal surface depends on its polar functional groups. Organic substances containing O, N, and/or S block active corrosion sites by adsorption on metal surface [137].

The process of corrosion inhibition by organic compounds typically involves two stages. First, the inhibitor migrates to the metal surface, where it interacts with substrate through adsorption. In the case of physisorption, its molecules do not form direct linkages with metals but are separated by a layer of solvent molecules already adsorbed on the surface [138,139].

Hadisaputra et al. [140] investigated how phenylphthalimide and its derivatives act as organic inhibitors against carbon steel corrosion. The study revealed that N, O and C atoms of the ring are the primary reaction sites, being capable of donating electrons to carbon steel surface. In aqueous solutions, all compounds tested were shown to vie with water to be adsorbed on metal surface.

Bahremand et al. [141] studied the synergistic inhibitory effects of nitrate (cerium III) and phosphate (trisodium) anions on low carbon steel corrosion in saline environments at three distinct mixing ratios. Electrochemical tests showed that the 500Ce–500TSP sample stimulated the formation of a strong covering layer on steel surface. This was evidenced by increased resistance to charge transfer and a reduction in corrosion current density, indicating a compact and adherent interfacial layer that limited electrolyte access to the metal. This layer improved corrosion resistance and its surface hydrophobicity, likely due to the presence of cerium hydroxide, contributed to effective corrosion inhibition and low wettability.

#### 2.5.2. Inorganic Corrosion Inhibitors

Inorganic corrosion inhibitors are substances added to corroding environments to form a protective film on metal surface aiming to slow or stop corrosion. Commonly utilized in various sectors, including the petroleum and gas, chemical, petrochemical, and civil construction ones, they can be categorized as anodic or cathodic inhibitors based on their chemical makeup and how they work [142].

Anode inhibitors, also called passivators, prevent the anodic reaction and, reacting with corrosion products, help create a cohesive, insoluble protecting film on metal surface. Examples of inorganic anode inhibitors are silicates, chromates, nitrates, phosphates, molybdates, and hydroxides [127].

Cathode inhibitors hinder cathode reaction during corrosion. Due to alkalinity, metal ions react to form insoluble compounds that precipitate at cathodic sites. Magnesium hydroxide [Mg(OH)_2_] and zinc hydroxide [Zn(OH)_2_] are common inorganic cathodic inhibitors that deposit at cathodic areas to protect the metal. Calcium salts and phosphonates are also used [143].

Aimini et al. [144] studied the release of lanthanum cations in piperazine-modified SBA-15 with the aim of inhibiting low carbon steel corrosion. Polarization experiments highlighted a 65% decrease in corrosion index for the lanthanum-containing sample after 1 day-immersion in NaCl solution compared to the reference.

#### 2.5.3. Green Corrosion Inhibitors

Traditional corrosion inhibitors are losing ground because of increased environmental concerns and changes in legislation, so ecofriendly corrosion inhibitors are increasingly sought after. Naturally occurring corrosion inhibitors are preferable for containing a broad spectrum of active components, which can be utilized in organic compounds to adhere to a metal surface and create a covering layer capable of preventing further corrosion. Since natural extracts are rich in such components, they represent a suitable source to develop novel green corrosion inhibitors. Various natural ingredients are being studied, along with their use in distinct applications, including steel reinforcement embedded into concrete [145,146,147].

Inorganic green inhibitors initiate the creation of an adsorption layer that tends to be fragile, making metal surface susceptible to pitting and crevice corrosions. Conversely, organic green inhibitors, when used in conjunction with inorganic inhibitors, are capable of passivating metal surface consistently and offer the maximum possible protection against the harsh environment [133]. Many types of green corrosion inhibitors, such as biopolymers, biosurfactants, surfactants, pharmaceuticals, chitosan, honey, yeast and botanical extracts, and amino acids, among others, are becoming popular for reducing corrosion in reinforced concrete constructions due to their availability, ecological friendliness, biodegradability, low toxicity, and renewable nature [148,149].

Environmentally friendly corrosion inhibitors have gained increasing interest in reinforced concrete structures because of their ability to protect embedded steel reinforcement while being environmentally compatible. In the alkaline solutions found in concrete pores, these inhibitors can adsorb on steel surface, creating protective films able to limit anode dissolution and cathode reactions. Additionally, some environmentally friendly inhibitors work by blocking concrete pores, reducing chloride ion penetration or binding to aggressive species, which delays the start of corrosion. Biopolymers, amino acids, plant extracts, and biosurfactants have demonstrated to enhance corrosion resistance by altering the steel–concrete interface and stabilizing the passive layer on the steel reinforcement [150].

Jebali et al. [151], through electrochemical analyses, demonstrated that the aqueous extract of *Ammophila arenaria* inhibited corrosion of mild steel by 83.67% (polarization) and 84.32% (impedance) in acidic environments. Focusing on corrosion inhibition power, Lakikza et al. [152], testing at room temperature *Alysicarpus compactum* extract as a natural inhibitor against St37 carbon steel corrosion in an acid medium (1 M HCl), reported an 85.48% peak inhibition efficiency at concentration of 0.4 g/L.

Therefore, to reduce the undesired impacts of corrosion, the adoption of appropriate monitoring methods and the implementation of some strategies are essential, such as design enhancement, anode and cathode protection, coatings and corrosion inhibitors. Due to their selectiveness and optimal performance, corrosion inhibitors appear to be the best option to combat corrosion [153].

Table 2 summarizes some representative examples, highlighting the diversity of compounds investigated, including organic, natural, and inorganic inhibitors. These results reinforce the importance of research on new inhibitory agents, considering not only anticorrosive efficiency but also factors such as cost, availability, and environmental impact.

Taking into consideration the pollution caused by corrosion inhibitors, one of the major industrial objectives is using nontoxic, ecofriendly, cheap substances. In this context, there is a growing interest in alternatives of natural origin that combine anti-corrosion efficiency with sustainability. Among these alternatives, biosurfactants stand out for being amphiphilic molecules of microbial origin that have shown great potential as ecological and economically viable inhibitors, constituting a promising field of research [168].

## 3. Biosurfactants

Compounds derived from bioprocesses performed by microorganisms, biosurfactants (BS), or natural surfactants have been increasingly recognized as a suitable alternative to synthetic surfactants because of their improved biocompatibility, biodegradability, and lower toxicity to the environment. These qualities make them more appealing from an ecological perspective. In many cases, biosurfactants have demonstrated reduced toxicity compared to conventional synthetic surfactants, especially at application-relevant concentrations; however, their environmental impact depends on molecular structure, dosage, and exposure conditions [169,170,171].

Microbial surfactants can be obtained from different substrates, including potato peelings, industrial residues, cassava flour, palm oil, raw glycerol, and soya oil. The lipophilic properties of BS come from long-chain fatty acids, whereas sugars, phosphates, carboxylic acids, amino acids, and cyclic peptides are responsible for their hydrophilicity [172,173].

These compounds can generally be divided into two categories: low- and high-molecular-weight BS. The former are more effective at lowering surface and interfacial tension, while the latter (bioemulsifiers) exhibit greater emulsifying activity [174].

Green surfactants offer several advantages over synthetic ones, such as easier biodegradability, ecofriendliness, higher foaming ability, specificity and effectiveness under extreme conditions (temperature, pH, and salinity), along with lower toxicity. For instance, biodegradability of a sophorolipid biosurfactant achieved 61% after 8-day culture, while sodium dodecyl sulfate (a synthetic surfactant) showed no biodegradability after 8 days [21,175].

Despite the advantages of their use, biosurfactants have a small market share due to high production costs. As a result, various alternatives have been explored to change this situation, including searching for low-cost raw materials like agro-industrial waste, optimizing culture media, developing extraction and purification technologies, and finding microorganisms with high productivity [176,177].

Biosurfactants today account for about 10% of global surfactant production and are utilized in many sectors including petroleum, food, pharmaceuticals, medicine, and agriculture [178].

Microorganisms that produce biosurfactants are often present in marine environments (algae species), land (sediments, soil, mud), extreme environments (oil reservoirs), foods (dairy, fermented foods, honey), and contaminated areas (wastewater, petroleum-polluted soil). They can survive across broad ranges of temperature, pH, and salinity [175].

Generally, biosurfactants are either anionic or nonionic. They are classified into two groups based on molecular weight: (i) low-molecular-weight surfactants (<1200 g/mol) and (ii) high-molecular-weight surfactants (>45,000 g/mol). The former are efficient at lowering interfacial tension, whereas the latter are better at stabilizing emulsions and foams [179]

### 3.1. Classification of Biosurfactants

BS are produced by a wide spectrum of different microorganisms as secondary extracellular compounds (in the stationary phase) that can either circulate in the culture medium or keep bound to cell surface. Moreover, the microorganisms that produce them can synthesize a broad range of biosurfactants based on their properties [180]. The main BS categories are glycolipids, lipopeptides, phospholipids, and polymeric surfactants [175].

#### 3.1.1. Glycolipids

Glycolipids are amphiphilic molecules because they contain both a hydrophilic glycosyl group and lipophilic lipid residues. In comparison to synthetic oil-based surfactants (such as alkylbenzene sulfonates) or plant-derived surfactants (like alkyl polyglycosides), glycolipid biosurfactants produced by microbes generally exhibit higher surface activity, better emulsifying ability, lower critical micelle concentrations, enhanced biodegradability, reduced ecotoxicity, and decreased protein denaturation capacity [181,182].

These BS are further divided into three major subcategories based on their polar tails: rhamnolipids, sophorolipids, and trehalolipids, among others. The most well-known microorganisms that produce glycolipids belong to the genera *Pseudomonas*, *Rhodococcus*, *Arthrobacter*, *Starmerella*, and *Candida* [183].

Glycolipids exhibit beneficial bioactive properties, such as antimicrobial, immunoregulatory and antiviral activities, thanks to which they are currently among the most widespread microbial BS globally [184].

Glycolipidic biosurfactants have been shown to cause growth stop and apoptosis in cancer cells, reinforcing their potential for clinical use. Additionally, they have demonstrated anticancer activity against various tumor cell lines, including the breast (MCF-7), colon (CaCo-2), liver (HepG2), and human promyelocytic leukemia ones. Specifically, the low toxicity of biosurfactants to humans, along with their high tolerance to extreme pH and temperature, makes them promise for medical use [185,186].

Sophorolipids, produced primarily by the yeast *Starmerella bombicola* (formerly *Candida bombicola*), do not exhibit cytotoxicity and are approved by the Food and Drug Administration for use in many industries [187].

#### 3.1.2. Lipopeptides

Lipopeptides are a new type of natural BS synthesized primarily by members of the genera *Bacillus*, *Streptomyces*, *Pseudomonas*, *Serratia*, *Aspergillus*, and *Actinomyces*. They are typically made of β-amino or β-hydroxy fatty acids (lipophilic groups) and peptide chains or peptide rings (hydrophilic groups) [188]. Their antibiofilm activity is mainly ascribed to water channel mechanism, whereas the antimicrobial one to their capability of disrupting cytoplasmic membranes, rising their permeability and leading to the loss of metabolites [189].

The main lipopeptides synthesized by *Bacillus* spp. belong to surfactin, iturin, and fengycin groups. They are grouped according to amino acid sequence, cyclization, peptide length, and fatty acid kind. The most common lipopeptides from *Pseudomonas* spp. are viscosin, amphisin, tolaasin, and syringomycin [190].

Currently, some reports have highlighted the role of lipopeptide BS in enhancing biohydrogen production through dark anaerobic fermentation of lignocellulosic biomass and their effects on the microbial ecosystem. Biosurfactants greatly improved biohydrogen production, which significantly influenced the generation of short-chain fatty acids [191,192].

#### 3.1.3. Phospholipids

Phospholipids are BS made up of a hydrophilic head with a phosphate and two hydrophobic fatty acid tails, commonly found in bacterial and yeast cytoplasmic membranes. They were studied for their capacity to lower interfacial tension and enhance oil recovery [193].

Phospholipids are fundamental constituents of the cytoplasmic membrane, mainly when saturated aliphatic hydrocarbons are employed as carbon sources for cultures. Such compounds, which are key to structure stability and functionality, are made up of a glycerol molecule with two fatty acids attached via ester bonds and a phosphate group, leading to various types of phospholipids depending on their components. Phosphatidylinositol is a typical example of phospholipid [194,195].

#### 3.1.4. Polymeric Biosurfactants

Polymeric BS are high-molecular-weight compounds produced by various species of the genera *Pseudomonas*, *Arthrobacter*, *Bacillus*, *Acinetobacter*, *Halomonas*, and *Candida*. Each kind of such BS has its own structure and features, enabling a broad variety of uses [194]]. The lipopolysaccharide known as Emulsan is a potent extracellular emulsifier produced from hydrocarbons by the bacterium *Acinetobacter calcoaceticus* [196].

To exploit at best BS potential, their interaction with different bio-based and man-made polymers has been investigated. Such interaction can result in special self-assemblies or complex structures with enhanced performance over the individual constituents [197]. A polymer can serve as a carrier or matrix for the biosurfactant, promoting its gradual controlled delivery. If combined with suitable polymers, they can create ecofriendly systems, which are especially beneficial in the controlled and sustained release of drugs. Such a synergy is also being investigated to improve petroleum recovery and environmental cleanup [198].

Because of their emulsifying capacity and capability of reducing liquid–solid interfacial tension, these BS can be used across various production segments, such as petroleum, cosmetics, food, and agriculture. Moreover, they are biodegradable and generally less toxic than the man-made counterparts, making them more ecosustainable and suitable for bioremediation efforts and helping remove pollutants and restore contaminated soils [199,200].

**Figure 5:**
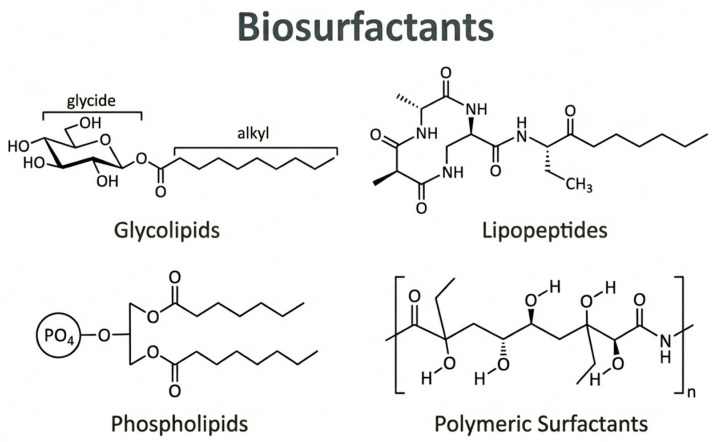
Main classes of biosurfactants and their representative chemical structures.

The chemical structure of biosurfactants varies greatly, leading to different physicochemical properties and potential uses, each with specific traits that affect their performance in industrial and environmental applications [201]. Figure 5 visually explains this classification.

The above classification highlights the wide structural diversity of BS, emphasizing that their chemical characteristics are directly related to their surface properties and application potential.

Although multiple studies report the corrosion inhibition effectiveness of various biosurfactants, a comparative analysis reveals distinct performance patterns among biosurfactant classes. Glycolipids, such as rhamnolipids and sophorolipids, typically show high adsorption efficiency at low concentration because of their amphiphilic nature and ability to form stable micelles under these conditions. Lipopeptides, in contrast, thanks to their greater film-forming ability and thermostability, are more appropriate for high-temperature conditions. Polymeric biosurfactants tend to provide longer-lasting protection, though they require higher dosages. These differences emphasize that corrosion inhibition effectiveness is influenced not only by biosurfactant concentration but also significantly by molecular structure, interfacial interactions, and environmental conditions [154].

### 3.2. Properties of Biosurfactants

Utilization of BS in different sectors takes advantage of their enhanced features over the traditional counterparts, including lower toxicity, greater biodegradability and environmental sustainability, better foaming property, action and tolerance to extreme conditions of temperature, salinity, and pH, and higher corrosion inhibition [202]. Below is a list of how some properties of BS work.

- Reduction in Surface Tension (ST): The ability to form micelles, i.e., clusters of amphiphilic molecules, is the main property of BS. A progressive increase in BS concentration reduces ST to a minimum value at which micelles form [203,204]. Thanks to their amphipathic structure, BS enable adsorption at the liquid interface, resulting in the formation of a monolayer that, reducing the molecular attraction between liquids, acts as a thin, elastic membrane; therefore, ST is reduced. BS can then lower ST between two substances, e.g., a gas and a liquid (surface tension of water), two liquids (water and oil), or a liquid and a solid (water and dirt particles) [205].
- Biodegradation is a removal mechanism that can be limited by low bioavailability of hydrophobic contaminants, such as petroleum hydrocarbons. However, bacteria can produce BS that can improve the bioremediation of hydrophobic compounds. A typical example is a microbial consortium that produced a sophorolipid that improved the bioaccessibility of petroleum hydrocarbons for bacterial degradation [206].
- Emulsification is a stable heterogenous disequilibrium process involving an immiscible liquid dispersed, in the form of small droplets, within another liquid. In agriculture, cosmetics, and pharmaceutical sectors, BS are usually employed as emulsifiers. Their emulsifying activity, which depends on substrate, allows their use to enhance bioremediation and remove petroleum from contaminated sites [207,208]. It is typically determined over time (e.g., 24, 96 and 168 h) to assess emulsion stability. The ratio of emulsion height to total liquid height is used as the emulsion index [209].
- Anti-biofilm activity: The use of BS as an alternative to prevent biofilm formation has been largely studied. The wettability and amphiphilic properties of BS enable them to affect corrosion by acting on the adhesivity of microbial cells. The biosurfactant lichenysin from *Bacillus licheniformis* demonstrated anti-biofilm activity either before or after treatment. Another example is provided by rhamnolipids, which exerted an anti-adhesive and anti-biofilm effect against Gram-positive and Gram-negative bacteria, inhibiting biofilm formation on polystyrene and stainless-steel surfaces [210].
- Antimicrobial action: Biosurfactants demonstrate antibacterial effects by disrupting cell membranes. Unlike traditional antibiotics, BS utilize alternative mechanisms to eliminate target microorganisms [211]. Green surfactants are notable for being low in toxicity and highly biodegradable, primarily because they are made up of simple sugars, fatty acids or polypeptides. Such features guarantee the safety of drug formulations, lower the risk of undesired side effects, and maintain the efficacy of bioactive compounds [212].
- Dispersion happens when the cohesive force among similar particles is reduced. Small quantities of dispersants like BS can be added to a suspension to avoid insoluble particle aggregation. For instance, BS can be used to remove lipophilic compounds from rock surface, making them more motile and facilitating their recover during petroleum extraction. Dispersion also plays a key role in diminishing or entirely suppressing the formation of undesired microbe biofilms [213].
- pH and temperature have little influence on the interface activity of most BS. The activity of lichenysin from *B. licheniformis* JF-2 was not affected in the pH range of 4.5 to 9.0 and at temperatures up to 50 °C. Likewise, a *Bacillus subtilis* LB5a lipopeptide kept its emulsifying activity for half a year at 121 °C, pH 4–10 and high salinity [10% (*w*/*v*) NaCl] [214].

#### Biosurfactants as Corrosion Inhibitors

BS effectively inhibit metal corrosion and exhibit very good adaptability to harsh alkaline, saline, or acidic conditions [215]. Due to their amphipathic nature, green surfactants are considered a major class of efficient organic corrosion inhibitors, mainly preferred in the field of corrosion prevention. Surfactants inhibit the corrosion of metals via adsorption pushed by electrostatic or electron-donating coordination with the hydrophilic moiety [216].

Adsorption of surfactants onto metallic surfaces effectively blocks electrochemically active sites, inhibiting corrosion. Polar functional groups, including hydroxyl, carboxyl, or amino, improve this process through interactions like electrostatic forces, hydrogen bonds, or coordination with metal surface [217]. Conversely, once adsorbed, the hydrophobic tails of surfactant molecules tend to orient outward from the metallic surface, promoting the formation of an organized and continuous hydrophobic layer. This layer works as a physical and chemical barrier capable of limiting the diffusion of water molecules, dissolved oxygen, and aggressive ionic species such as chlorides or sulfates toward the metal–solution interface [218]. Hence, the mass transfer processes essential for corrosion reactions are significantly hindered, leading to an overall improvement in anticorrosive performance [219].

**Figure 6:**
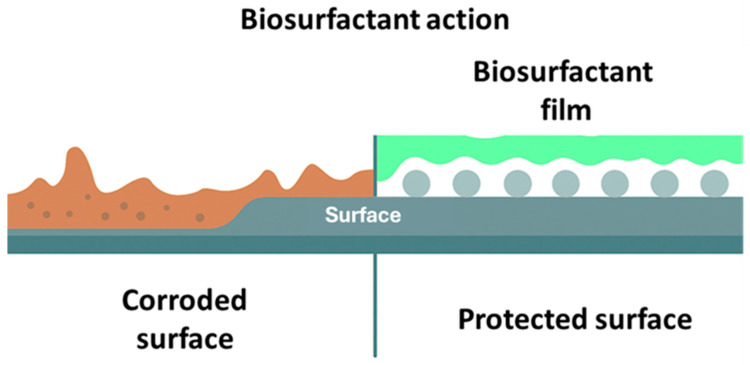
Mechanism of action of a biosurfactant as a metal surface inhibitor.

Figure 6 illustrates the mechanism of action of BS on metallic surfaces.

BS are able to lower the surface tension of a metal and enhance its wetting properties. The critical micelle concentration (CMC) is the point where surfactant molecules provide the maximum surface coverage and corrosion protection [220,221]. Beyond such a concentration, BS begin to aggregate, forming micelles. Therefore, increasing biosurfactant concentration beyond the CMC can sometimes negatively affect their anticorrosive properties [222].

Although natural BS can act as efficient metal corrosion inhibitors, their extraction and purification are complex and expensive, limiting their practical use in corrosion protection. Chemically synthesized BS offer a promising solution to this issue. Both natural and synthetic BS are capable of interacting with carbon steel surface through electron interaction with unoccupied *d* orbitals, leading to strong adsorption and demonstrating anti-corrosive properties. Being effective in preventing metal corrosion, it is believed that both natural and synthetic BS represent the future of ideal corrosion inhibitors aligned with the principles of green chemistry [216].

Recently, the number of patents on biosurfactant-based corrosion inhibitors has increased significantly, reflecting growing industrial interest in sustainable, environmentally friendly solutions. Table 3 lists a compilation of these patents, highlighting the types of BS studied, the metallic substrates involved, and the corrosive media used in the investigations.

Analysis of patents reveals the growing technological interest in using BS as corrosion inhibitors, reflecting the advancement of research aimed at replacing synthetic compounds with sustainable alternatives. In this context, a promising scenario for the development of new formulations and industrial applications can be envisioned, as reinforced by studies focused on improving the stability, efficiency, and economic viability of these compounds presented below.

Olivia et al. [232] investigated the potential of a biosurfactant from *Penicillium citrinum* in corrosion inhibition, comparing it to the synthetic surfactant Tween 80 (P8074) using solid steel bars submerged in 0.9% NaCl. Samples treated with the biosurfactant showed less mass loss when compared to those treated with the synthetic surfactant. Iravani et al. [233] investigated the efficiency of two oleic acid-based BS, namely sorbitol oleate and pentaerythritol oleate, in reducing acid corrosion of carbon steel in water saturated with H_2_S-CO_2_ produced from oil fields. Both compounds effectively inhibited corrosion, since the addition of 24 × 10^−4^ M sorbitol and pentaerythritol oleates remarkably reduced the corrosion rate (to 0.005 and 0.033 mm y^−1^) compared to the control (0.490 mm y^−1^), corresponding to inhibition efficiency of 98.9 and 93.3%, respectively. Selva Filho et al. [4], who evaluated the incorporation of a *S. bombicola* ATCC 222214 biosurfactant in a biodegradable matrix as a corrosion inhibitor, observed a significant reduction in corrosion induced by atmospheric physical factors in carbon steel samples. After one-month exposure in an Accelerated Corrosion Chamber, the mass losses per unit area were 123.60 g/m^2^ in the control and 25.22, 18.93 and 22.12 g/m^2^ using the biosurfactant at concentrations equivalent to half the CMC, the CMC and twice the CMC, respectively, confirming the high efficiency of the biosurfactant-incorporating matrix.

In light of the above, a promising trend can be observed for the application of BS in mitigating metallic corrosion in different productive sectors, especially given the growing demand for innovative and technologically efficient and environmentally sustainable solutions.

## 4. Conclusions

This review critically examined the use of biosurfactants as corrosion inhibitors, focusing on their physicochemical properties, adsorption behavior, and inhibition mechanisms reported in various corrosive environments. The studies analyzed show that biosurfactants can effectively decrease corrosion rate through surface adsorption, forming protective interfacial films, and in some cases, altering biofilm activity. Their performance heavily depends on molecular structure, concentration, and environmental conditions.

Compared to traditional synthetic inhibitors, biosurfactants offer promising benefits related to biodegradability and potentially lower environmental impact; however, their efficiency, stability, and durability are highly dependent on formulation. Main challenges identified include scaling up production, cost-effectiveness, long-term stability under extreme conditions, and absence of standardized methods for performance assessment.

Future research should focus on structure–performance relationships, advanced surface characterization techniques, and the development of hybrid or multifunctional inhibitor systems to improve protection efficiency and industrial use. Overall, biosurfactants are a viable and promising alternative for sustainable corrosion control, though not universally applicable.

## Figures and Tables

**Table 1:** Synthesis of studies on corrosion mechanisms.

**Corrosion Type**	**Material/Environment Studied**	**Aim of the Study**	**Reference**
**General or uniform corrosion**	Pipelines in petroleum and gas sector	Optimization of Weibull distribution for risk calculation	[66]
	Marine structural steel	Installation of high-strength E690 steel corrugated sandwich panel	[67]
**Bimetallic or galvanic corrosion (GC)**	ADC12 Aluminum alloy and copper	Study of GC between ADC12 alloy and Cu in a 3.5% (*w*/*w*) NaCl solution	[68]
	Screw joints	Study of GC of an Al coating in contact with a 55Al-Zn coating in an atmospheric environment	[69]
**Pitting corrosion (PC)**	Reinforcing bars	Evaluation of the tensile behavior of reinforcing bars with simulated pitting corrosion	[70]
	Duplex stainless steel 2205	Investigation of the effect of tensile elastic stress on PC mechanism and passive film of steel in a sulfide-containing NaCl solution	[71]
**Crevice corrosion (CC)**	90/10 Cu-Ni alloy	Evaluation of alloy susceptibility to CC caused by *Desulfovibrio vulgaris*	[72]
	X70 Pipeline steel	Study of electroactive *Shewanella* sp. in accelerating steel CC in a marine environment	[73]
**Intergranular corrosion (IC)**	Superaustenitic stainless steel	Study of the effect of molybdenum on steel IC	[74]
	Sensitized type 304 stainless steel	Study of the role of MnS in steel IC and depassivation	[75]
**Erosion corrosion (EC)**	As-cast TC4 cast and LPBF TC4	Study of cavitation erosion corrosion properties of as-cast TC4 and LPBF TC4 in 0.6 mol/L NaCl solution	[76]
	Ni_2_FeCrMo_0.2_HEA	Evaluation of the effect of sand particle size on alloy resistance to EC in seawater	[77]
**Stress corrosion cracking (SCC)**	Austenitic stainless steel	Study of the influence of grain size and boundary type on steel intergranular SCC	[78]
	2205 Duplex stainless steel	Study of the behavior and corrosion mechanism of steel under applied polarization potential	[79]

**Table 2:** Synthesis of studies on corrosion inhibitors, highlighting compounds, materials, environments, methodologies employed, and results obtained.

Inhibitors	Compound	Material	Corrosive Medium	Results	Reference
**Organic**	Benzimidazole with tryptophan units	Carbon steel	1.0 M HCl	Inhibition efficiency (IE) achieved 86.68%	[154]
	3-Chloro-4-morpholin-4-yl-1,2,5-thiadiazole	Mild steel	1.0 M HCl	The compound significantly reduced mild steel corrosion, reaching 96% IE	[155]
	2,4-Dinitrophenyl-hydrazine	Aluminum	1.0 M HCl	The compound proved to be a highly efficient inhibitor, with 88.9% IE	[156]
	11-(2-Chlorophenyl)-3,3-dimethyl-2,3,4,5,10,11-hexahydro-1H-dibenzo[be][1,4]diazepin-1-one	Carbon steel	1.0 M HCl	IE reached 94.4%	[157]
**Inorganic**	Lignin phosphate	Carbon steel	3.5% (*w*/*w*) NaCl	The corrosion rate was reduced by approximately 54.2%	[158]
	Sodium phosphate (Na_3_PO_4_)	Carbon steel	0.6 M Cl^−^	IE reached 91.7%	[159]
	Sodium phosphate and sodium dodecyl sulfate	Magnesium AZ91	3.5% (*w*/*w*) NaCl	The use of the two compounds together resulted in an initial IE of 99.7%	[160]
	Phytic acid and sodium phosphate	SLM 304L steel	3.5% (*w*/*w*) NaCl	The synergistic effect allowed for a maximum IE of 92.35%	[161]
**Green**	Leaf extract of *Andrographis paniculata*	Mild steel with the following composition (% by weight): 0.38 Si, 0.42 Cr, 0.12 P, 0.26 Ni, 0.43 Mn, 0.12 C, 0.42 Cu	H_2_SO_4_	The extract, at a concentration of 4000 ppm, ensured an IE of 95.14%	[162]
	Extract from *Cassia fistula* leaves	Carbon steel in simulated concrete pore solution	0.5 mol/L NaCl	The results revealed a maximum IE of 85.21% (from mass loss) and 92.4% (from potentiodynamic polarization)	[163]
	Powder from the bark of *Punica granatum*	Carbon steel bars in reinforced concrete structures	3.5% (*w*/*w*) NaCl	The mass loss test revealed a maximum IE of 83.11%	[164]
	Extract from reflux rubber seeds	Cold-rolled steel	1.0 M HCl	The extract achieved an IE of 95.09%	[165]
	Biosurfactant from *Pseudomonas cepacia* CCT6659	Carbon steel	Abiotic and biotic systems	The biosurfactant IE was 80 and 87% for abiotic and biotic systems, respectively	[166]
	Rhamnolipid	Carbon steel X70	Simulated seawater	The corrosion rate was reduced by approximately 72.2%	[167]

**Table 3:** Patents registered on biosurfactants used in the chemistry and metallurgy sectors as corrosion inhibitors, specifying types of biosurfactants, metallic substrates, and corrosive media.

NO.	Patent Number	Patent Title	Description	Year	Reference
1	US2016237334A1	Method of using biosurfactants as acid corrosion inhibitors in well treatment operations	The corrosive effects resulting from well treatment applications are inhibited and/or prevented by introducing into the well a formulation containing a selected biosurfactant as a corrosion inhibitor	2016	[206,223]
2	CN114645279A	Application of rhamnolipid as environment-friendly microbial corrosion inhibitor	The present invention describes the application of a rhamnolipid as a green biocorrosion inhibitor and belongs to the technical field of microbial corrosion protection. The rhamnolipid is used to inhibit metal material biocorrosion	2022	[207,224]
3	EP4098726A1	Use of at least one amphipathic biosurfactant as an alkaline corrosion inhibitor	The present invention describes a water-based alkaline preparation containing at least one glycolipid biosurfactant and at least one water-insoluble lubricant, and to its use in processing fluids	2022	[225]
4	US12065613B2	Multifunctional composition for enhanced oil recovery, improved oil quality and prevention of corrosion	The present invention discloses compositions and methods for concurrently improving oil recovery and quality by reducing sulfur-bearing substances, and hindering or lowering corrosion of oil and gas production equipment. A multifunctional composition is provided, comprising an antimicrobial biosurfactant component, an ammonium salt, and ammonium hydroxide	2024	[226]
5	CN117165947A	Medical instrument antirust agent based on organic corrosion inhibitor and preparation method of medical instrument antirust agent	The invention describes an antirust agent for medical instruments based on an organic corrosion inhibitor and its method of preparation. The rust inhibitor is prepared from an organic corrosion inhibitor (phospholipid), a dispersant, a biosurfactant, an auxiliary agent, and deionized water	2023	[227]
6	CN117363429A	Cleaning agent containing sophorolipid as well as preparation method and application of cleaning agent	The invention describes a sophorolipid-based cleaning agent, with excellent properties such as cleaning power, corrosion inhibition, sterilization, and bacteriostasis, which can perform effective cleaning and protection functions on automotive windshields and similar surfaces	2024	[228]
7	US2025075118A1	Biosurfactants for iron and zinc sulfide scale remediation and control	The present invention describes scale inhibitors and/or dispersants comprising biosurfactants, as well as methods of their use to remove scale deposits	2025	[229]
8	CN112680289A	Aviation engine carbon deposit cleaning agent and preparation method thereof	The present invention describes a cleaning agent for carbon deposits in aviation engines using a sophorolipid biosurfactant, among other components, to protect all engine assemblies against corrosion, oxidation, and discoloration. At the same time, the agent promises to completely remove carbon deposits and dirt from all engine assemblies, ensuring effective cleaning	2021	[230]
9	CN119040907A	Bio-based efficient cleaning rust remover material and preparation method thereof	The present invention, which belongs to the technical field of metal surface treatment, provides an efficient biologically based rust remover and a method for preparing it	2024	[231]

## Data Availability

No new data were created or analyzed in this study. Data sharing is not applicable to this article.
